# Application of mTORC1 Inhibitors for Tissue-Agnostic Management of Standard-Therapy-Refractory Solid Tumors

**DOI:** 10.3390/cancers14081936

**Published:** 2022-04-12

**Authors:** Hossein Taghizadeh, Agnieszka Maj-Hes, Gerald W. Prager, Leonhard Müllauer, Robert M. Mader

**Affiliations:** 1 Department of Medicine I, Medical University of Vienna, 1090 Vienna, Austria; seyed.taghizadehwaghefi@meduniwien.ac.at (H.T.); agnieszka.maj-hes@meduniwien.ac.at (A.M.-H.); gerald.prager@meduniwien.ac.at (G.W.P.); 2 Comprehensive Cancer Center Vienna, 1090 Vienna, Austria; leonhard.muellauer@meduniwien.ac.at; 3 Department of Medicine I, University Hospital St. Poelten, Karl Landsteiner University of Health Sciences, 3100 St. Poelten, Austria; 4 Clinical Institute of Pathology, Medical University Vienna, 1090 Vienna, Austria

**Keywords:** targeted therapy, molecular oncology, mTOR, tissue-agnostic

## Abstract

**Simple Summary:**

Hyperactivation of the mTOR pathway is a common occurrence in malignancies. This study investigated the clinical benefit of the tissue-agnostic application of mTOR inhibitors for the therapeutic management of a pan-cancer cohort of patients with mTOR pathway aberrations. Seventy-one patients were offered the targeted therapy and twenty-three eventually received it. Only three patients (4.2%) achieved stable disease, of whom one experienced progressive disease again after 9.1 months. Thus, in selected patients with heavily pretreated solid tumors with activation of the mTOR pathway, the antitumoral activity of mTORC1 inhibition was weak.

**Abstract:**

In this analysis, we examined the efficacy, feasibility, and limitations of the application of mTOR inhibitors based on the individual molecular profiles of pretreated cancer patients after the failure of all standard treatments in the palliative setting. In this single-center, real-world analysis of our platform for precision medicine, we analyzed the molecular characteristics of 71 cancer patients. The tumor samples of the patients were analyzed using next-generation sequencing panels of mutation hotspots, microsatellite stability testing, and immunohistochemistry. All profiles were reviewed by a multidisciplinary team to provide a targeted treatment recommendation after a consensus discussion. Seventy-one cancer patients with activation of the mTOR pathway were offered an mTORC1-inhibitor-based targeted therapy, and twenty-three (32.4%) of them eventually received the targeted therapy. Only three patients (4.2%) achieved stable disease, of whom one experienced progressive disease again after 9.1 months. The median time to treatment failure was 2.8 months. In total, 110 mutations were detected in 60 patients (84.5%). The three most frequent mutations were found in *TP53*, *PTEN*, and *KRAS*, which accounted for over 50% (56.4%) of all mutations. In sum, in selected patients with heavily pretreated solid tumors with activation of the mTOR pathway, the antitumoral activity of mTORC1 inhibition was weak.

## 1. Introduction

The serine/threonine-specific protein kinase mammalian target of rapamycin (mTOR) is encoded by *MTOR* and belongs to the family of phosphatidylinositol 3-kinase (PI3K)-related kinases. mTOR forms two protein complexes together with other proteins, referred to as mTOR complex 1 (mTORC1) and mTOR complex 2 (mTORC2). mTOR acts as a core catalytic subunit of these two complexes and plays a fundamental role in cell growth and proliferation.

mTOR plays a fundamental role in cell physiology, particularly through its downstream effectors 4EBP1 and P70S6 kinase (S6K), as it coordinates vital cell processes including cell growth and proliferation [1].

The mTOR pathway is embedded in the PI3K/Akt/mTOR signaling pathway. Hyperactivation of the mTOR pathway is a common occurrence in malignancies as it promotes carcinogenesis and cancer growth. This pathologic pathway activation can either occur downstream or be caused by the inactivation or deletion of mTOR suppressors, including tuberous sclerosis 1 (*TSC1*), tuberous sclerosis 2 (*TSC2*), and phosphatase and tensin homolog (*PTEN*). Thus, targeting mTOR may be an effective antitumoral therapeutic strategy [2,3,4].

The mTORC1 inhibitors everolimus and temsirolimus are orally administered immunosuppressive and antiproliferative drugs that inhibit the PI3K/Akt/mTOR signaling pathway. These drugs form a complex with the cyclophilin FKBP-12 that binds to the mTOR subunit of mTORC1. Thus, everolimus and temsirolimus block downstream signaling and attenuate cell growth and proliferation.

Thus far, both the Food and Drug Administration (FDA) and European Medicines Agency (EMA) have approved everolimus for the therapeutic management of hormone-receptor (HR)-positive, human epidermal growth factor receptor 2 (HER2)-negative breast cancer (combined with exemestane); pancreatic neuroendocrine tumors; renal cell carcinoma (RCC); and the tuberous sclerosis complex (TSC) [5]. Temsirolimus has been approved for the treatment of RCC by the FDA and EMA and for mantle cell lymphoma (MCL) only by the EMA [6,7].

In this analysis, we investigated the tissue-agnostic application of mTORC1 inhibitors for the management of therapy-refractory solid tumors.

## 2. Methods

### 2.1. Patients and Design of the Precision Medicine Platform

The precision medicine platform of the Comprehensive Cancer Center of the Medical University of Vienna (CCC-MUV) was open to patients with various heavily pretreated metastatic cancers who had progressed through all standard treatment options as determined by response evaluation criteria in solid tumors 1.1 (RECIST 1.1), provided tissue samples for molecular profiling were available. If tumor biopsy was not feasible, specimens from the Department of Pathology’s archives were used as replacements. The performance status of patients had to be 0 or 1 according to the Eastern Cooperative Oncology Group (ECOG). The precision medicine platform provides targeted therapy recommendations to patients with therapy-refractory solid tumors. For this analysis, patients had to provide informed consent before inclusion in the platform as well as be at least 18 years old at the time of molecular analysis. The Institutional Ethics Committee of the Medical University of Vienna (Nr. 1039/2017) approved this analysis by consensus. If no other guideline-based therapy regimens were available for the tumor patients, the General Hospital of Vienna directly covered the costs of the molecular analysis and administration of the targeted therapy.

### 2.2. Tissue Samples

Formalin-fixed, paraffin-embedded (FFPE) tissue samples from patients with metastatic cancers that had advanced beyond the scope of standard therapy regimens were obtained from the archives of the Department of Pathology, Medical University of Vienna, Austria.

### 2.3. Cancer Gene Panel Sequencing

DNA was extracted from either FFPE tissue blocks or fresh biopsies using the QIAamp Tissue Kit^TM^ (Qiagen, Hilden, Germany). DNA sequencing was conducted on 10 ng of DNA per tissue block. DNA libraries were generated by multiplex polymerase chain reaction (PCR) using the Ion AmpliSeq Cancer Hotspot Panel v2 (Thermo Fisher Scientific, Waltham, MA, USA), which is composed of 50 mutation hotspots covering driver mutations, tumor suppressor genes, and oncogenes. In mid-2018, the gene panel was expanded using the 161-gene next-generation sequencing (NGS) panel of the Oncomine Comprehensive Assay v3 (Thermo Fisher Scientific) that covers genetic alterations and gene fusions. The Ampliseq Cancer Hotspot Panel was sequenced with an Ion PGM sequencer (Thermo Fisher Scientific) and the Oncomine Comprehensive Assay v3 with an Ion S5 sequencer (Thermo Fisher Scientific). Afterward, the generated sequencing data were analyzed with the help of the Ion Reporter Software (Thermo Fisher Scientific). BRCA Exchange, ClinVar, COSMIC, dbSNP, OMIM, and 1000 Genomes were referred to for variant calling and classification. The variants were classified according to a five-tier system comprising the modifiers pathogenic, likely pathogenic, uncertain significance, likely benign, and benign. This classification was based on the standards and guidelines for the interpretation of sequence variants of the American College of Medical Genetics and Genomics [8]. In recommending targeted therapy, pathogenic and likely pathogenic variants were considered.

### 2.4. Immunohistochemistry (IHC)

A Ventana BenchMark Ultra stainer (Ventana Medical Systems, Tucson, AZ, USA) was used to perform IHC on tissue sections with a thickness of 2 µm. A variety of antibodies were added, including epidermal growth factor receptor (EGFR; clone 3C6; Ventana Medical Systems), HER2 (clone 4B5; Ventana Medical Systems), HER3 (clone SP71; Abcam, Cambridge, UK), mTOR (clone 49F9; Cell Signaling Technology, Danvers, MA, USA), programmed death-ligand 1 (PD-L1; clone E1L3N; Cell Signaling Technology (until mid-2018; as of mid-2018, Nordic Biosite, Stockholm, Sweden, is using the BSR90 clone)), and PTEN (clone Y184; Abcam).

To assess the immunostaining intensity for the antigens EGFR, mTOR, PDGFRA, PDGFRB, and PTEN, a combinative semi-quantitative score was used. For a comprehensive description of the IHC, we refer to our previous work [9].

### 2.5. FISH

PTEN loss was verified only with FISH in selected cases. FFPE sections of 4 μm thickness were used for FISH along with the following probe: PTEN (10q23.31)/Centromere 10 (ZytoVision, Bremen, Germany). Approximately 200 cells per tumor were examined. Approximately 30% of cells with only one or no PTEN signal were considered positive for *PTEN* gene loss on the PTEN FISH. FISH analysis of the centromere of chromosome 10 was used to control for ploidy.

### 2.6. Multidisciplinary Team (MDT) for Precision Medicine

An experienced molecular pathologist examined the molecular profiles of each tumor sample, followed by analysis by an MDT.

In addition to molecular pathologists, radiologists, clinical oncologists, and surgical oncologists, the MDT included basic scientists. Each patient’s molecular profile was combined with pathological parameters to generate a targeted treatment recommendation. Tyrosine kinase inhibitors, checkpoint inhibitors (e.g., anti-PD-L1), and antibodies targeting growth factor receptors with or without endocrine therapy were among the targeted therapies. Based on phase I through phase III clinical trials, the MDT prioritized treatment recommendations based primarily on the level of evidence.

A therapeutic regimen targeting as many molecular driver aberrations as possible was recommended for patients with more than one druggable molecular aberration, with specific consideration of each antitumor drug’s toxicity profile and interaction. Before being included in our precision medicine platform, all patients received a full spectrum of standard treatment options for their cancer condition, including off-label use of nearly all targeted agents. Patients who qualified in terms of their tumor profile and clinical characteristics were asked to enroll in a clinical trial conducted at our cancer center, which recruited patients for targeted therapies if they showed a willingness to take part in that particular trial.

### 2.7. Study Design and Statistics

Overall survival (OS) and progression-free survival (PFS) were analyzed using IBM SPSS Statistics software version 25 and presented using Kaplan–Meier curves. Data were presented using medians, and frequency distributions were used to delineate the characteristics of the patients with metastatic solid tumors. Statistical significance was defined as a *p*-value of less than 0.05. For statistical calculations, the software package IBM SPSS Statistics version 26 was employed.

## 3. Results

### 3.1. Patient Characteristics

From the initiation of our platform for precision medicine in June 2013 until June 2021, 554 patients with therapy-refractory cancer were included in our platform for precision oncology. Of these 554 patients, we identified 71 patients with different solid tumors with no further standard treatment option available who were all recommended an mTORC1-inhibitor-based therapy based on the activation of the mTOR pathway. All 71 patients were Caucasian and included 44 women (62.0%) and 27 men (38.0%).

The patient cohort comprised 13 different tumor entities with gynecologic malignancies constituting the largest one (Table 1). The median age at first diagnosis was 56.4 years, and the median age at the time of molecular profiling was 60.6 years (Table 1). In 44 cases (62.0%), fresh tumor tissue was obtained for the generation of a current molecular profile by biopsy in 35 patients (49.3%) and during surgical treatment in 9 patients (12.7%). In the other 27 cases, archived FFPE tissue was used for the creation of the molecular portrait. 

In the 44 abovementioned cases, the median time period between tissue collection and review by the MDT and therapy initiation (for the 23 patients who were treated with an mTORC1-inhibitor-based therapy) was 36 and 44 days, respectively. In cases where FFPE was employed, the time interval between tissue preservation and therapy initiation was 13.6 months.

Disease relapse had occurred in 45 of the patients who previously underwent curative surgical resection. Metastases were documented in all patients, primarily in the lymph nodes, liver, lungs, and bones. 

Peritoneal and pleural carcinomatosis was diagnosed in 18 (25.4%) and 4 patients (5.6%), respectively. The patients were administered a median of three lines of prior palliative therapy ranging from two to seven lines. Prior to molecular profiling, 41 patients (57.7%) were treated with at least three lines of palliative anticancer treatment.

### 3.2. Molecular Profile

In 26 cases (36.6%), the molecular profile was created from a tumor specimen of the primary tumor site. In the remaining 45 cases (63.4%), the profile was generated from a secondary tumor site, mainly from affected lymph nodes (*n* = 26). Fifty-five samples were analyzed by the Oncomine Comprehensive Assay v3, and sixteen specimens were tested by the Ion AmpliSeq Cancer Hotspot Panel v2. 

A total of 110 mutations in 60 patients (84.5%) were observed. Genetic aberrations were most commonly documented for *TP53* (*n* = 34; 47.9%), *PTEN* (*n* = 15; 21.1%), and *KRAS* (*n* = 13; 18.3%), making up over half (56.4%) of all aberrations. Three patients who did not receive the mTORC1-inhibitor-based therapy harbored an *STK11* mutation. We did not observe any concordance between *STK* mutations and the IHC score of mTOR. 

For 11 (15.5%) patients, we could not observe any genetic aberrations (Table 2). In our cohort, we did not detect any genetic aberrations in *TSC1*, *TSC2*, or *MTOR*. 

Three gene fusions were identified in two patients, including PTPRK-RSPO3 in one patient and TBL1XR1-PIK3CA and ESR1-CCDC170 in the other patient.

Moreover, we detected five different gene amplifications in two different tumor specimens, including *MYC* (*n* = 2), *PDGFRA*, *KIT*, *KRAS*, and *RICTOR*.

**Figure 1:**
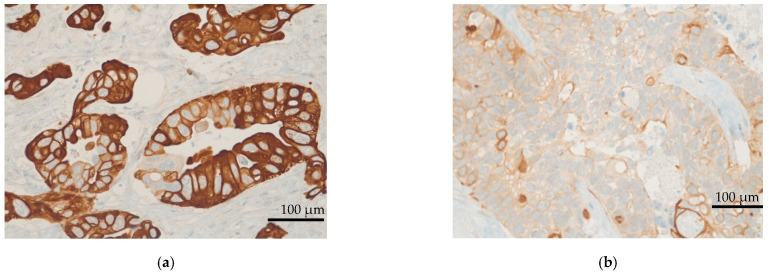

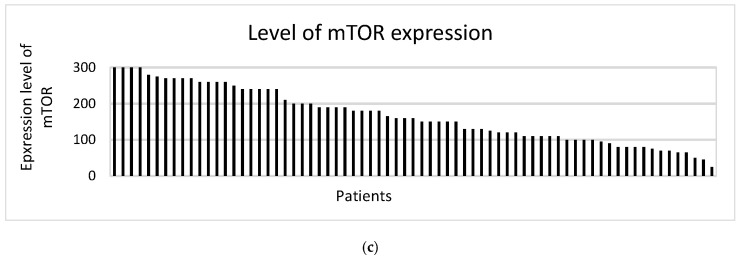
(**a**) Representative image of high expression of mTOR (IHC score = 300). (**b**) Representative image of low expression of mTOR. (Images by kind courtesy of Prof. Dr. Müllauer). (**c**) Level of mTOR expression in 71 patients with therapy−refractory solid tumors. Scale bar = 100 μm.

**Figure 2:**
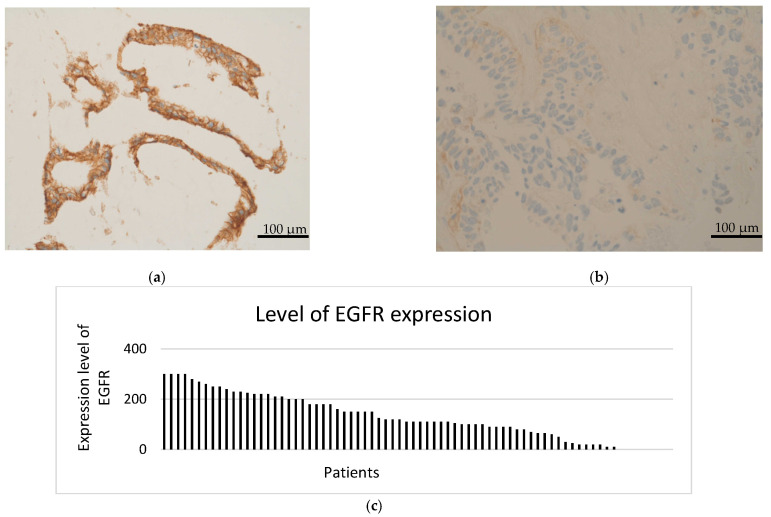
(**a**) Representative image of high expression of EGFR (IHC score = 300). (**b**) Representative image of low expression of EGFR. (Images by kind courtesy of Prof. Dr. Müllauer). (**c**) Level of EGFR expression in 71 patients with therapy−refractory solid tumors. Scale bar = 100 μm.

The mTOR pathway was activated in all patients, with high scores of mTOR expression in IHC (between 200 and 300) in 24 patients (33.8%). The median IHC score of mTOR was 160 (Figure 1 and Figure 2).

**Figure 3:**
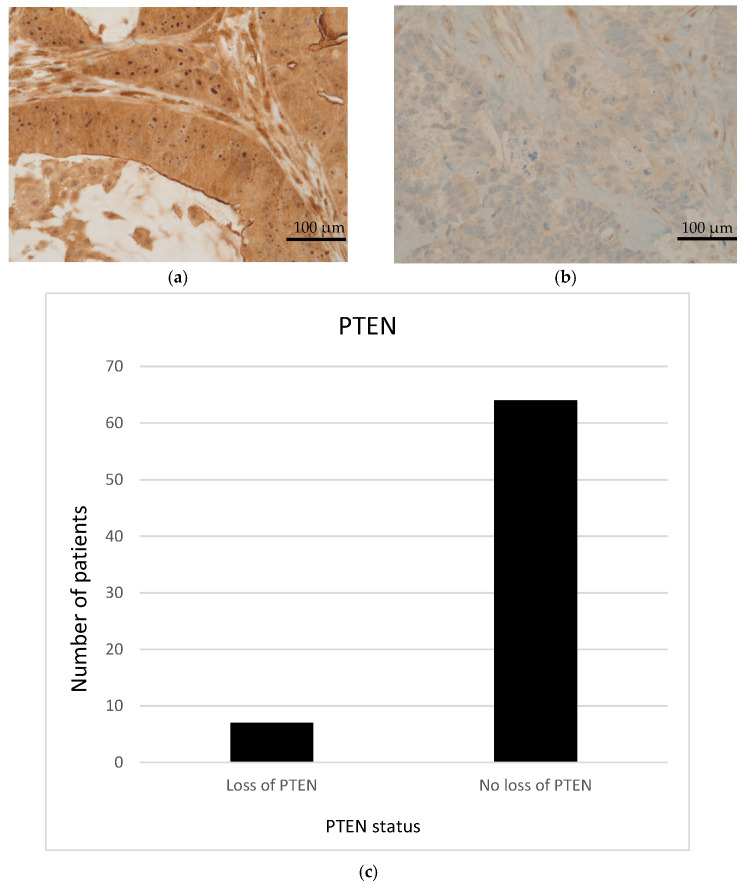
(**a**) Representative image of high expression of PTEN (IHC score = 300). (**b**) Representative image of low expression of PTEN. (Images by kind courtesy of Prof. Dr. Müllauer). (**c**) Loss of PTEN in 9 out of 71 patients with therapy−refractory solid tumors. Scale bar = 100 μm.

**Figure 4:**
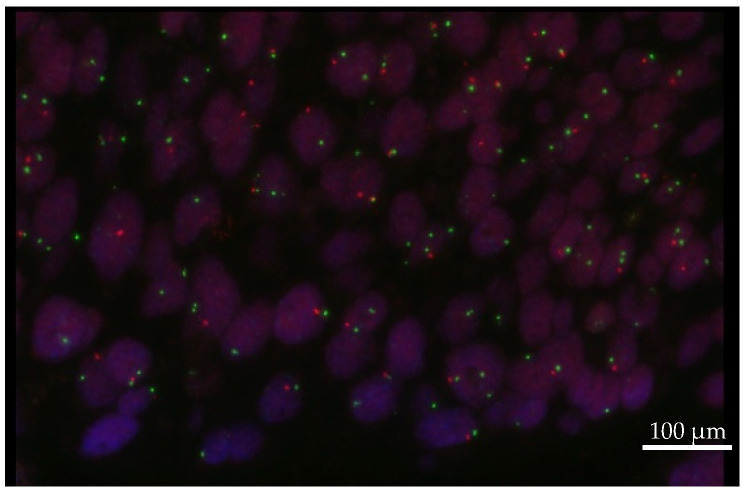
FISH image of heterozygous loss of PTEN. The red signal represents PTEN (chromosome location 10q23), and the green signal represents centromere 10. In the case of a heterozygous deletion, one red signal (10q23) is accompanied by two green signals in the nucleus. (Image by kind courtesy of Prof. Dr. Müllauer). Scale bar = 100 μm.

Moreover, IHC demonstrated EGFR expression in 52 (73.2%) patients. The median EGFR score was 110. Nineteen patients (26.8%) displayed high EGFR expression (scores between 200 and 300; Figure 3 and Figure 4). Seven patients (9.9%) appeared to have a loss of PTEN in IHC, which was subsequently verified by FISH as heterozygous PTEN deletions (Figure 1, Figure 2, Figure 3 and Figure 4). Further, MET (*n* = 31; 43.7%) and PDGFRA (*n* = 14; 19.7%) were also frequently expressed. None of the patients had a status of MSI-H. NGS could not be performed for one female patient due to insufficient tumor material. 

### 3.3. Therapy Recommendations and Outcome

In the majority of cases, everolimus (*n* = 67; 94.4%) was recommended, whereas temsirolimus was only recommended in four patients (5.6%), of whom three were diagnosed with head and neck squamous cell carcinomas and one with carotid paraganglioma.

Of the 67 cases (94.4%), everolimus was recommended as monotherapy for 36 patients (50.7%) and in combination with another anticancer agent in the other 31 cases (43.7%). The two most important combination agents were exemestane (*n* = 21; 29.6%) and cetuximab (*n* = 8; 11.3%). Exemestane was predominantly recommended for the therapy of gynecologic malignancies (*n* = 19; 26.8%) harboring a strong expression of the estrogen receptor. Cetuximab was offered to patients with a distinct EGFR expression (Table 3).

In this work, the vast majority of the targeted therapy recommendations (*n* = 69; 97.2%) were entirely derived from the molecular characteristics determined by IHC. Only in two cases (2.8%), for which imatinib and sorafenib were recommended, did the findings from NGS help to shape the clinical decision. 

In total, 23 patients (32.4%) were treated with the mTORC1-inhibitor-based therapy. Five patients (7.0%) died before the radiological reassessment of therapeutic response. Two female patients (2.8%) treated with everolimus combined with exemestane discontinued the therapy due to intolerable skin toxicity.

Eventually, radiological restaging was performed in 16 patients (22.5%; Table 4). Thirteen patients (18.3%) did not respond to treatment and had progressive disease. In three patients (4.2%), a stabilization of the disease course was observed. Thus, the disease control rate was 4.2% (see Table 3 and Table 4 for further information). At the time of data cutoff, one of the three patients had already experienced progressive disease again. The two other patients were still treated with the mTORC1-inhibitor-based therapy.

**Figure 5:**
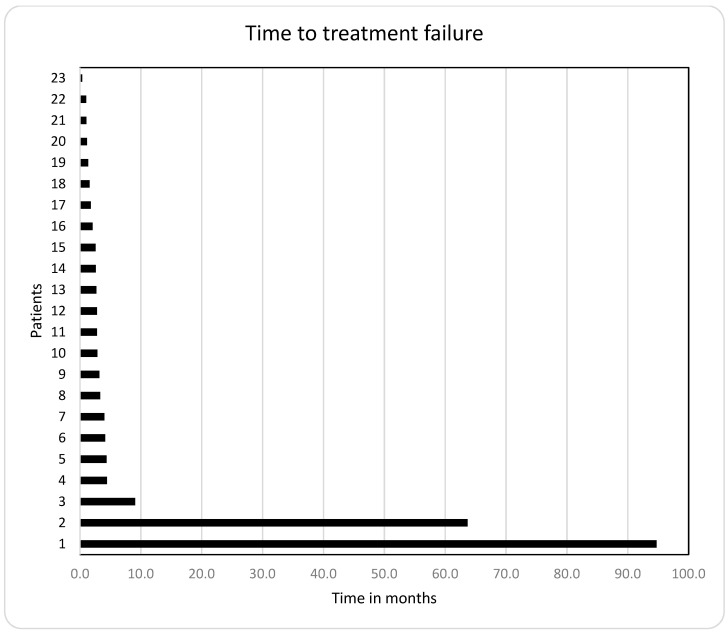
Time to treatment failure (TTF) in 23 cancer patients who received mTORC1-inhibitor-based therapy: the median TTF was 2.8 months.

The median time to treatment failure (TTF) in the 23 patients who were treated with the mTORC1-inhibitor-based therapy was 2.8 months (0.4–94.7 months; Figure 5 and Table 4). 

**Figure 6:**
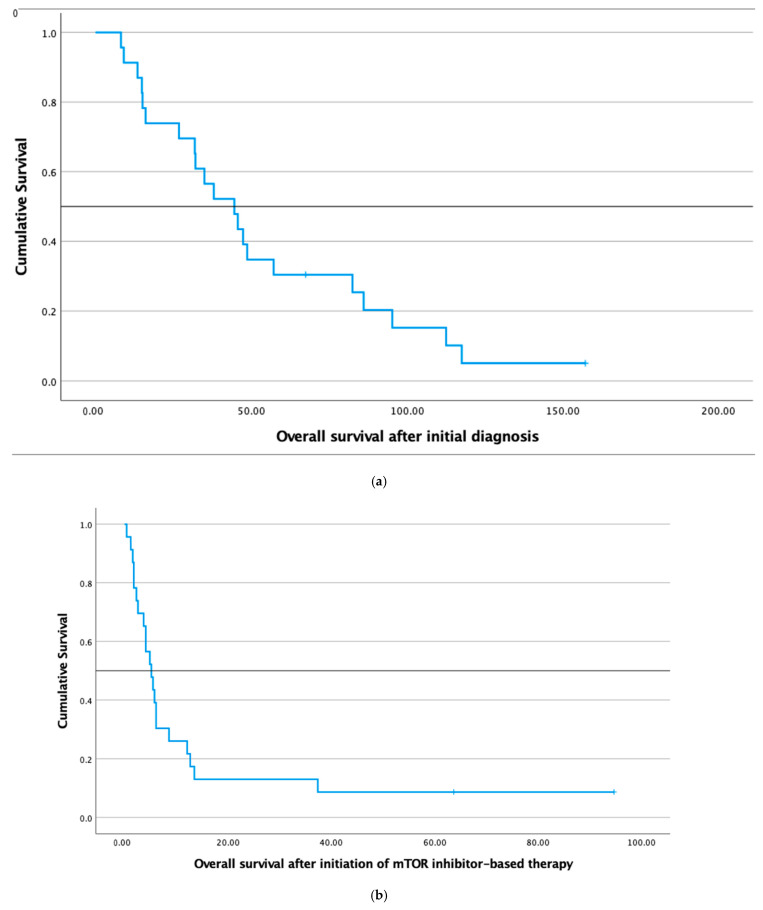
(**a**) Kaplan–Meier survival curve demonstrating overall survival (OS) after initial diagnosis of the solid tumor and (**b**) Kaplan–Meier survival curve showing OS after the beginning of the mTORC1-inhibitor-based therapy in 23 patients.

The median overall survival (mOS) of these 23 patients after the initial diagnosis of the malignancy was 44.6 months. The mOS after initiation of targeted therapy was 5.2 months (see Figure 6).

**Figure 7:**
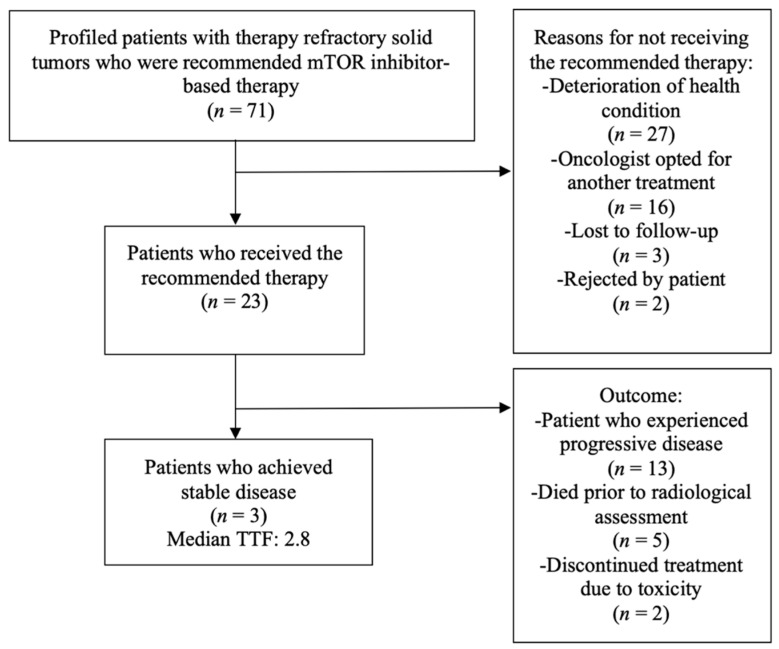
Flow chart of the 71 cancer patients.

Three patients (4.2%) were lost to follow-up after the suggestion of the molecular-driven targeted therapy. In total, 45 patients (63.4%) were not treated with the mTORC1-inhibitor-based therapy for different reasons, including rapid deterioration of the general health condition (*n* = 27; 38.0%), preference of the treating oncologist for an alternative treatment (*n* = 16; 22.5%), and refusal of further treatments by the patient (*n* = 2; 2.8%). Figure 7 depicts the patient flow.

## 4. Discussion

In this study, we recommended 71 mTOR-inhibitor-based therapies for pretreated cancer patients primarily based on the strong activation of the mTOR pathway. In nearly all cases, the targeted therapy recommendations with mTORC1-inhibitor-based therapy were primarily based on the molecular information gained from IHC. This finding underscores the importance of IHC for the recommendation of targeted therapies.

It is worth mentioning that out of 30 female patients with gynecologic malignancies, 19 patients had a significant expression of mTOR and the estrogen receptor. Thus, in these cases, everolimus was offered in combination with exemestane based on the BOLERO-2 phase 3 trial that led to the approval of this combination therapy for patients with advanced hormone-receptor-positive/HER2-negative breast cancer who progressed with prior nonsteroidal aromatase inhibitor therapy [10]. In this phase 3 trial, the median PFS was 6.9 months with everolimus plus exemestane versus 2.8 months with the placebo plus exemestane. Response rates were 9.5% and 0.4% in the combination therapy and exemestane-alone groups, respectively.

Another important phase 3 trial, the ARCC trial, tested the efficacy of temsirolimus versus interferon and combination therapy with temsirolimus and interferon in over 600 patients with previously untreated metastatic RCC. Patients who received temsirolimus alone had a superior OS (10.9 months) and PFS (3.8 months) when compared with patients who received interferon alone or combination therapy. Temsirolimus achieved an objective response rate of 8.6% [11].

In four cases, temsirolimus was recommended (in two cases as monotherapy and in two cases in combination with another drug) for patients with HNSCC based on several phase 2 trials that showed the antitumor activity of temsirolimus in this entity [12,13,14]. The half-life of temsirolimus is half that of everolimus [15]. Everolimus was not suggested for HNSCC since it did not appear to be effective in this tumor entity in a phase 2 trial [16].

The disease control rate (DCR) in our cohort was below 5%, with only three patients achieving stable disease out of twenty-three patients who received the recommended targeted therapy. Of these three patients, one patient experienced progressive disease after 9.1 months. Thus, the antitumoral activity of mTOR-inhibitor-based therapies in pretreated tumor patients was weak. Our study indicates that the activation of the mTOR pathway alone may not be a suitable predictive marker for therapeutic response to mTOR-inhibitor-based therapy. 

Our findings are in line with a phase 2 basket trial by Adib et al. that investigated the therapeutic activity of everolimus in selected patients with different advanced solid tumors who harbored *TSC1/TSC2* or *MTOR* mutations [17]. Adib et al. reported that everolimus had a disappointing objective response rate (7%), with a median PFS of 2.3 months and median OS of 7.3 months. The trial did not exclude pretreated patients; however, it did not specify how many patients were pretreated and how many lines of therapy they received before trial inclusion. Adib et al. did not perform IHC. We performed genomic profiling to evaluate the influence of mutations in upstream and downstream effectors of the mTOR pathway on the efficacy of the mTOR-inhibitor-based therapy. In our cohort, we did not detect any genetic aberrations in *TSC1*, *TSC2*, or *MTOR*. Three patients who did not receive the mTOR-inhibitor-based therapy harbored an *STK11* mutation. We did not observe any concordance between *STK* mutations and the IHC score of mTOR.

One possible explanation for the poor clinical outcome may be that, similar to rapamycin, temsirolimus and everolimus do not suppress the main downstream mTOR effectors S6K and 4EB-P1 equally effectively: S6K is more sensitive to mTOR inhibitors than 4EB-P1 [18]. In addition, the unsatisfactory results seen in our cohort are due to partial inhibition by mTORC1 inhibitors leaves residual mTOR activity and possible alternative activation routes via mTORC2 signaling. It is proven that mTORC2 plays a pivotal role in cancer metabolic reprogramming and carcinogenesis [19,20]. 

Another explanation may be that the mTOR pathway interacts with other compensatory pathways and thus may circumvent inhibition by everolimus by several negative feedback loops via the activation of other signaling pathways, such as MAPK signaling, for tumor proliferation. This may include aberrations in the RAS pathway and downstream ERK signaling [2,21,22]. In line with this explanation, we found 13 mutations in KRAS and 4 aberrations in BRAF. Apart from that, O’Reilly et al. showed that mTOR inhibition itself can induce insulin receptor substrate-1 expression and abrogate feedback inhibition of the pathway, resulting in upstream activation of Akt [22].

*TP53*, *PTEN*, and *KRAS* mutations together made up more than half of all mutations found.

Except for the *KRAS* G12C mutation, there are still no approved targeted therapies that are aimed at genetic aberrations in *KRAS*, *PTEN*, and *TP53*. Targeting these mutations is a clinical need that is yet to be met. Other mutations were of low prevalence (less than 10%), mirroring tumor heterogeneity.

Strikingly, nearly 40% of the patients (*n* = 27) did not receive the recommended targeted therapy due to the rapid deterioration of their health condition. We assume that this is because of the long median turnaround time. For the 23 patients who received the mTOR-inhibitor-based therapy, the time period between tissue collection and review by the MDT and the beginning of molecular-based therapy was more than a month. 

In precision oncology, the turnaround time is a crucial aspect that must be taken into consideration. A turnaround time of over 1 month without any adequate anticancer treatment will result in disease progression. Progressive metastases, in particular in the liver, may culminate in metastatic liver failure and rapid health deterioration, making any further treatment attempt impossible. As a result of the long turnaround time, it is possible that the time was insufficient for the targeted therapy to be fully effective.

Our study has several limitations. The molecular profile was primarily created based on two techniques: genetic profiling and IHC. However, the molecular portrait of malignant tissues is complex and multilayered and may not be covered by these two techniques. A thorough analysis of the molecular tumor portrait includes many other dimensions, such as genomics, epigenomics, transcriptomics, proteomics, metabolomics, and microbiomics [23]. Integration of such massive data volumes and their translation into precision oncology is a major undertaking. Moreover, we did not investigate the expression and activity of downstream targets of mTORC1, such as S6RP and 4E-BP1.

Moreover, this study may be subject to several limitations, including bias in selection, incomplete recording of clinical information, insufficient attention to possible confounders, and nonexistent randomization.

Another limitation is that in 27 cases, archived FFPE tissue was used to generate a molecular portrait with a turnaround time between tissue fixation and the initiation of molecular profiling of 13.6 months. Tumor biology is highly dynamic, changes over time, and is shaped and sculpted by the antitumoral therapy itself [24,25,26]. A median time delay of over 13 months means that the molecular landscape may have changed in this period and the molecular map generated from the FFPE samples may not have represented the current status of the cancer’s biology and mutational burden. These circumstances may lead to the limited efficacy of mTOR-inhibitor-based therapy and may explain the modest activity of mTOR inhibitors in our cohort to some extent.

In 26 cases (36.6%), the molecular profile was created from a tumor specimen of the primary tumor site. In the remaining 45 cases (63.4%), the profile was generated from a secondary tumor site, mainly from affected lymph nodes (*n* = 26). Tumors are characterized by a highly dynamic and complex molecular intratumoral and intertumoral heterogeneity that changes both temporally and spatially [23,27,28,29,30,31]. Therefore, the biopsy from the metastasis may not reflect the molecular characteristics of the primary tumor.

Liquid biopsy may be a solution to both reduce the long turnaround time of over 1 month from the biopsy of the tumor tissue to the completion of the molecular profile and to provide a comprehensive cross-sectional molecular landscape representing the extreme tumor heterogeneity [32,33].

## 5. Conclusions

Taken together, the tissue-agnostic therapeutic management of a pan-cancer cohort of patients with mTOR pathway aberrations posed several major challenges, including the weak activity of mTOR inhibitors and the long turnaround time. A profound understanding of the abnormal mTOR pathway and its upstream and downstream regulators is essential to augment the efficacy of inhibitors of this pathway and minimize the occurrence of therapeutic resistance.

## Figures and Tables

**Table 1:** Patient characteristics (*n* = 71).

Patient Characteristics	Number
Median (range) age in years at first diagnosis	56.4 (17.7–76.9)
Median (range) age in years at molecular profiling	60.6 (19.3–80.3)
Female patients	44 (62.0%)
Male patients	27 (38.0%)
Caucasian	71 (100%)
Relapsed cancer	45
Metastatic cancer	71 (100%)
Systemic anticancer treatment received	71 (100%)
Prior lines of systemic anticancer treatment	2–7
mTORC1-inhibitor-based therapy applied: • in female patients; • in male patients.	23 (32.4%)
	16 (22.5%)
	7 (9.9%)
Tumor entities	
Gynecologic malignancies	30 (42.3%)
Colorectal cancer	7 (9.9%)
Head and neck squamous cell carcinomas	6 (8.5%)
T-cell lymphoblastic lymphoma	2 (2.8%)
Prostate cancer	3 (4.2%)
Pancreatic ductal adenocarcinoma	2 (3.8%)
Tumors of the central nervous system	2 (3.8%)
Biliary tract cancer	3 (4.2%)
Fibrolamellar hepatocellular carcinoma	2 (3.8%)
Cancer of unknown primary	2 (3.8%)
Gastroesophageal junction cancer	3 (4.2%)
Non-small-cell lung carcinoma	4 (5.6%)
Neuroendocrine neoplasms	5 (7.0%)
Mutations detected relevant to the PI3K/Akt/mTOR signaling pathway	
PTEN	15
PIK3CA	4
STK11	3
AKT1	2
MTOR	0
TSC1	0
TSC2	0
Total number of mutations detected	110

**Table 2:** Genomic profile of the therapy-refractory solid tumors (*n* = 71).

Mutated Genes	Number of Mutations	Percentage of Occurrence in Patients (*n* = 71)	Percentage of All Mutations (*n* = 110)
*TP53*	34	47.9	30.9
*PTEN*	15	21.1	13.6
*KRAS*	13	18.3	11.8
*APC*	4	5.6	3.6
*BRAF*	4	5.6	3.6
*KIT*	4	5.6	3.6
*PIK3CA*	4	5.6	3.6
*ARID1A*	3	4.2	2.7
*CTNNB1*	3	4.2	2.7
*IDH1*	3	4.2	2.7
*STK11*	3	4.2	2.7
*AKT1*	2	2.8	1.8
*BRCA2*	2	2.8	1.8
*NF1*	2	2.8	1.8
*NRAS*	2	2.8	1.8
*ATM*	1	1.4	0.9
*ATR*	1	1.4	0.9
*ATRX*	1	1.4	0.9
*CHD1*	1	1.4	0.9
*CDKN2A*	1	1.4	0.9
*EGFR*	1	1.4	0.9
*ERBB2*	1	1.4	0.9
*PIK3R1*	1	1.4	0.9
*PTPN11*	1	1.4	0.9
*RB1*	1	1.4	0.9
*RET*	1	1.4	0.9
*SMARCA4*	1	1.4	0.9
Total	110	-	100

**Table 3:** Rationale for targeted therapy recommendations.

Therapeutic Agent (Trade Name)	Targets	Overview of Current FDA Approval for Different Entities	Overview of Current EMA Approval for Different Entities	Number of Recommended and Received Cases and Responses
Everolimus monotherapy	mTORC1	HER2-negative and hormone-receptor-positive advanced breast cancer, pancreatic neuroendocrine tumors, RCC, renal angiomyolipoma, and subependymal giant cell astrocytomas (SEGAs) with tuberous sclerosis complex (TSC)	Breast cancer, RCC, and Neuroendocrine tumors of pancreatic, gastrointestinal, or lung origin	Recommended for 36 patients with strong mTOR expression 7 patients received the therapy: 1 patient achieved stable disease 2 patients died prior to radiological assessment 4 patients experienced progressive disease
Cetuximab	EGFR	CRC and HNSCC	CRC and HNSCC	Recommended in combination with everolimus for 8 patients with EGFR expression and strong mTOR expression 4 patients received the therapy: 1 patient achieved stable disease 1 patient died prior to radiological assessment 2 patients experienced progressive disease Recommended in combination with temsirolimus for and applied in 2 patients with head and neck squamous cell carcinomas with EGFR expression and strong mTOR expression: 1 patient died prior to radiological assessment 1 patient experienced progressive disease
Exemestane	Aromatase	Estrogen-receptor-positive breast cancer	Estrogen-receptor positive breast cancer	Recommended in combination with everolimus for 21 patients with estrogen expression and strong mTOR expression 8 patients received the therapy: 2 patients discontinued therapy due to toxicity 1 patient died prior to radiological assessment 5 patients experienced progressive disease
Sorafenib	PDGFR, RAF kinase, VEGFR,	HCC, RCC, and thyroid carcinoma	HCC, RCC, and thyroid carcinoma	Recommended in combination with everolimus for and applied in 1 patient with KIT expression, PDGFRA expression, and strong mTOR expression who experienced progressive disease
Imatinib	ABL1, BCR, KIT, and PDGFR	Ph+ CML, KIT+ GIST, MDS/MPD associated with PDGFR, and Ph+ ALL	Ph+ CML, KIT+ GIST, MDS/MPD associated with PDGFR, and Ph+ ALL	Recommended in combination with everolimus for 1 patient with KIT mutation, PDGFRA expression, and strong mTOR expression
Temsirolimus monotherapy	mTOR	RCC	MCL and RCC	Recommended for 2 patients with strong mTOR expression 1 patient received the therapy and achieved stable disease

ABL1—Abelson murine leukemia viral oncogene homolog 1; AML—acute myeloid leukemia; ALL—acute lymphatic leukemia; BCR—breakpoint cluster region; CML—chronic myeloid leukemia; CRC—colorectal cancer; EGFR—epidermal growth factor receptor; EMA—European Medicines Agency; FDA—Food and Drug Administration; GIST—gastrointestinal stromal tumor; HER2—human epidermal growth factor receptor 2; HNSCC—head and neck squamous cell carcinoma; MCL—mantle cell lymphoma; MDS—myelodysplastic syndrome; MPD—myeloproliferative disorder; PDGFR—platelet-derived growth factor receptor; Ph+—Philadelphia chromosome-positive; mTOR—mammalian target of rapamycin; RCC—renal cell carcinoma; VEGFR—vascular endothelial growth factor.

**Table 4:** Characteristics of the cancer patients receiving mTOR-inhibitor-based therapy (*n* = 23).

Number, Gender, Tumor entity	Detected Mutations, Gene Fusions, FISH	Score in Immunohistochemistry	Applied Targeted Therapy	Age (Years) at Molecular Profiling	TTF (Months)	Therapeutic Response	Cause of Therapy Termination
1 Male Fibrolamellar hepatocellular carcinoma	*AKT1*	EGFR = 300, PTEN = 220, mTOR = 270	Everolimus + cetuximab	29.7	94.7	SD	n.a.* (Therapy ongoing)
2 Female Carotid paraganglioma	*TP53*	mTOR = 200	Temsirolimus monotherapy	51.5	63.7	SD	n.a.* (Therapy ongoing)
3 Male Sigmoid colon cancer	*NRAS:* c.C181A *PTEN:* c.T302G	EGFR = 220, mTOR = 100	Everolimus monotherapy	74.7	9.1	SD	PD
4 Female Cancer of unknown primary	*AKT1:* c.G49A *SMAD4:* c.G1051C	EGFR = 150, MET = 2, mTOR = 110	Everolimus monotherapy	77.4	4.4	PD	PD
5 Female Endometrial cancer	*PTEN:* c.G389A	EGFR = 20, ER (Allred score) = 3, PR (Allred score) = 9 mTOR = 270	Everolimus + exemestane	61.3	4.4	PD	PD
6 Female Biliary tract cancer	*BRAF:* c.G1397T	MET = 2, mTOR = 100, ER (Allred score) = 3, PR (Allred score) = 6	Everolimus + exemestane	65.5	4.2	PD	PD
7 Female Ovarian cancer	*TP53:* c.759_767del *CHD1:* c.1467_1487del	EGFR = 80, ER (Allred score) = 12, PR (Allred score) = 3, PTEN = 140, mTOR = 125	Everolimus + exemestane	53.7	4.0	PD	PD
8 Male T-cell lymphoblastic lymphoma	*PTEN:* c.696del, heterozygous deletion detected by FISH	mTOR = 80	Everolimus monotherapy	21.6	3.3	PD	PD
9 Male Tongue cancer	PTEN: *c.C301T*, heterozygous PTEN deletion detected by FISH	EGFR = 210, mTOR = 150, MET = 1	Temsirolimus + cetuximab	59.4	3.2	PD	PD
10 Female Cervical cancer	*TP53:* c.G1015T,	EGFR = 100, ER (Allred score) = 8, PR (Allred score) = 8, mTOR = 70	Everolimus + exemestane	38.9	2.9	PD	PD
11 Female Sigmoid colon cancer	*TP53*: c.G626A, heterozygous PTEN deletion detected by FISH	EGFR = 110, MET = 1, mTOR = 240	Everolimus + cetuximab	19.3	2.8	PD	PD
12 Male Prostate cancer	No mutations detected, heterozygous PTEN deletion detected by FISH	mTOR = 110	Everolimus monotherapy	69.7	2.8	PD	PD
13 Male Rectal cancer	*TP53*: c.G743GA *KRAS:* c.A182AT, heterozygous PTEN deletion detected by FISH	EGFR = 50, mTOR = 65	Everolimus monotherapy	56.9	2.7	PD	PD
14 Male Biliary tract cancer	*BRAF:* c.G1397A *CDKN2A:* c.G256A	EGFR = 250, MET = 3, mTOR = 200	Everolimus + cetuximab	60.8	2.6	PD	PD
15 Female Ovarian cancer	*TP53:* c.G800A	EGFR = 20, mTOR = 300, ER (Allred score) = 8, PR (Allred score) = 6	Everolimus + exemestane	64.7	2.6	n.a.	Died
16 Female Ovarian cancer	*TP53:* c.815T > A	EGFR = 10, PTEN = 80, mTOR = 180, ER (Allred score) = 7, PR (Allred score) = 5	Everolimus + exemestane	61.8	2.1	PD	PD
17 Female Ovarian cancer	*TP53: c.681dup*, heterozygous deletion detected by FISH	MET = 2, PTEN = 140	Everolimus monotherapy	71.9	1.8	n.a.	Died
18 Male Prostate cancer	*IDH1: c.C394T*,	EGFR = 260, MET = 1, mTOR = 240	Everolimus + cetuximab	59.7	1.6	n.a.	Died
19 Male T-cell lymphoblastic lymphoma	No mutations detected	KIT = 3, mTOR = 200	Everolimus + sorafenib	21.4	1.4	PD	PD
20 Female Gastric neuroendocrine tumor	No mutations detected	mTOR = 180	Everolimus monotherapy	74.7	1.2	n.a.	Died
21 Female Ovarian cancer	*TP53: c.C742T,* TBL1XR1–PIK3CA gene fusion, ESR1–CCDC170 gene fusion	EGFR = 230, ER (Allred score) = 7, PTEN = 70, mTOR = 110	Everolimus + exemestane	77.9	1.1	n.a.	Toxicity
22 Female Ovarian cancer	*PIK3R1: c.C1106T*, *TP53: c.A1789T*, *NF1: c.C8070A*	ER (Allred score) = 7, PTEN = 150, mTOR = 180	Everolimus + exemestane	55.2	1.0	n.a.	Toxicity
23 Male Squamous cell carcinoma of the retromolar trigone	*CTNNB1: c.C172T*, heterozygous deletion detected by FISH	EGFR = 240, mTOR = 50	Temsirolimus + cetuximab	64.3	0.4	n.a.	Died

AR—androgen receptor; CPS—combined prognostic score; ECOG PS—Eastern Cooperative Oncology Group performance status; EGFR—epidermal growth factor receptor; ER—estrogen receptor; MSI-H—microsatellite instability-high; n.a.*—not applicable; PD—progressive disease; PD-L1—programmed death-ligand 1; PDGFRA—platelet-derived growth factor receptor alpha; mTOR—mammalian target of rapamycin; PR—progesterone receptor; SD—stable disease; PTEN—phosphatase and tensin homolog; TPS—tumor-positive score.

## Data Availability

Data are contained within the article.
